# Potential protective association of the AA genotype and a allele of CXCR4 rs2228014 polymorphism with COVID-19 severity in adult egyptians

**DOI:** 10.1186/s12879-024-09602-8

**Published:** 2024-10-15

**Authors:** Osama H. Korayem, Amr E. Ahmed, Mohamed H. Meabed, Doaa M. Magdy, Wafaa M. Abdelghany

**Affiliations:** 1 Biotechnology and Life Sciences Department, Faculty of Postgraduate Studies for Advanced Sciences, Beni-Suef University, Beni-Suef, Egypt; 2 Department of Pediatrics, Faculty of Medicine, Beni-Suef University, Beni-Suef, Egypt; 3 Department of Chest Disease and Tuberculosis, Faculty of Medicine, Assiut University, Assiut, Egypt; 4 Department of Clinical and Chemical Pathology, Faculty of Medicine, Cairo University, Cairo, Egypt

**Keywords:** *CXCR4*, COVID-19, SARS-CoV-2, Chemokines, Severe, Mild

## Abstract

**Background:**

By the end of December 2019, a new coronavirus, termed severe acute respiratory syndrome coronavirus 2 (SARS-CoV-2), emerged, and the cause of the disease was named coronavirus disease 2019 (COVID-19). Several genetic factors have been implicated in diverse responses to SARS-CoV-2 infection, such as the C-X-C chemokine receptor 4 (*CXCR4*) rs2228014 polymorphism, which has been previously studied in various diseases but has not been explored in the context of COVID-19 severity. The current study aimed to assess the association between the rs2228014 polymorphism in the *CXCR4* gene and the severity of COVID-19, which has not been previously reported.

**Method:**

This cross-sectional study analyzed 300 adult Egyptian COVID-19 patients (156 with mild or moderate and 144 with severe or critical symptoms) admitted to Assiut University Quarantine Hospital from June to September 2022 during the omicron variant. The rs2228014 polymorphism in the *CXCR4* gene was detected using real-time PCR with a TaqMan assay probe. Receiver operating characteristic (ROC) curve analysis was used to determine the best cutoff values for C-reactive protein (CRP) that can be used to estimate the severity of COVID-19. P values less than 0.05 were considered to indicate statistical significance.

**Results:**

No significant differences in the allelic or genotypic frequencies of *CXCR4* rs2228014 were detected between the severity groups. However, the exclusive presence of the AA genotype in mild or moderate cases suggests its potential protective role. Additionally, significant differences in myalgia presentation, leukocyte counts and antibiotic use, were observed among different genotypes. Statistical data showed that the severity of COVID-19 could be predicted at a cutoff value of CRP > 30 mg/L, with a sensitivity of 74.3% and a specificity of 42.9%.

**Conclusion:**

The present findings suggest a potential protective role of the AA genotype and A allele of *CXCR4* rs2228014 against severe COVID-19. Additionally, factors such as lack of vaccination and comorbidities such as hypertension, renal disease, and diabetes mellitus were associated with increased disease severity.

**Supplementary Information:**

The online version contains supplementary material available at 10.1186/s12879-024-09602-8.

## Introduction

By the end of December 2019, a scenario similar to the 1918 flu epidemic was emerged. Due to developments in medical research, it has been determined that this new infectious agent belongs to the coronavirus family. Quick genome sequencing efforts by many organizations have helped determine the composition and capabilities of the virus, as well as its immunogenicity across a range of demographics and possible defense mechanisms [1].

This novel virus is termed severe acute respiratory syndrome coronavirus 2 (SARS-CoV-2), and the associated disease is termed coronavirus disease 2019 (COVID-19) [2]. By the 22nd of January 2023, the virus caused more than 664 million confirmed cases and more than 6.7 million deaths worldwide [3]. In May 2021, the World health of organization (WHO) declared terminologies for categorization of SARS-CoV-2 emerging variants as Alpha, delta and Omicron. Omicron variant has come out through November 2021. It is considered the highest mutant variant with about 50 mutations. This variant characterized by high infection rate, rapid transmission with variance in the presenting symptoms that tend to be milder than previous ones [4].

Improved diagnostic methods supported by bioinformatics techniques are contributing significantly to the current SARS-CoV-2 pandemic. Bioinformatics has contributed to the rapid development of numerous diagnostic tools and methodologies for SARS-CoV-2 genome detection via next-generation sequencing. Because of the identification of COVID-19 biomarkers, these advances have also improved therapeutic strategies. Bioinformatics-based medication repurposing techniques to treat this deadly sickness have been developed [5].

COVID-19 is a respiratory infection that manifests as a disease similar to dengue fever, such as fever, exhaustion, a severe headache, and dry coughing [6]. People with SARS-CoV-2 infection experience a variety of signs and symptoms, ranging from mild illness to severe illness. Critical patients may develop severe organ damage, such as heart attack, acute kidney failure, liver dysfunction, and acute respiratory distress syndrome, which can cause an ongoing decrease in pulmonary function that may lead to death [7].

There are many factors that may contribute to the severity of COVID-19. First, there were factors such as obesity, advanced age, and male sex. Second, people with chronic diseases such as diabetes, chronic degenerative diseases, and kidney and heart diseases were excluded. The third factor is genetic predisposing factors [8].

Two genome-wide significant loci were identified in the first genome-wide association study (GWAS) of COVID-19 clinical outcomes. These loci mapped to the 3p21.31 region, which encompasses six genes (SLC6A26, LZTFL1, CXCR6, CCR1, CCR3, and CCR9), and to the 9q34.2 region, which contains the ABO blood group locus. The study compared 1,980 patients with severe disease from Italy and Spain with population controls of unknown SARS-CoV-2 infection status [9].

The COVID-19 Host Genomics Initiative (HGI) has identified a number of additional variants and genes linked to the severity or susceptibility to infection, including loci involving innate immunity or inflammation, such as the tyrosine kinase 2 (TYK2) crucial for antiviral responses, the interferon alpha and beta receptor subunit 2 gene (IFNAR2), the inflammasome regulator Dipeptidyl peptidase 9 (DPP9), and the oligoadenylate synthetase OAS1/OAS2/OAS3 gene cluster encoding activators of antiviral restriction enzymes [10].

Non-coding RNAs (ncRNAs) are transcripts found in the genome that are not meant to be translated into proteins. More than 98% of the RNAs present in human cells are comprised of them. However, a growing body of evidence indicates that certain ncRNAs are essential for controlling the expression of particular genes, which implies that they can modify gene expression in a transmissible, adaptive, and reversible way without altering the DNA sequence. Some ncRNAs have been linked to certain medical illnesses, including cancer, heart disease, neurological disorders, developmental disorders, and serious infectious diseases including HIV/AIDS and TB [11].

Chemokines are derivatives of cytokines, and they are named chemokines due to their chemotactic role in different immune cells. Chemokines and their receptors are considered to play central roles in many physiological processes, such as immune reactions, wound curing, and cancer formation. Chemokines affect adaptive and innate immune responses. They play a role in the inflammatory process by mediating the chemotaxis of white blood cells, which causes neutrophil and monocyte recruitment to where tissue injury or infection occurs [12].

Chemokines are expressed on all types of leukocytes and can be categorized into two groups: atypical chemokine receptors (ACKRs) and conventional chemokine receptors (CKRs) [13].

C-X-C chemokine receptor 4 (*CXCR4*) is mostly expressed by both normal and cancerous cells of hematopoietic or nonhmatopoietic ancestry. C-X-C chemokine ligand 12 (*CXCL12*) is considered the only ligand that can bind to *CXCR4*. In *CXCR4* knockout mice, this receptor has significant effects on immune cell organization, development, and hematopoiesis. *CXCL12/CXCR4* gene deletion in mice results in a fatal phenotype characterized by defective myeloid cell production and B lymphocyte production and irregular cardiovascular and neuronal system development [14].

The *CXCR4* gene is located on the long arm of chromosome 2 at position 21, while rs2228014 is located on exon 2 of *CXCR4* [15].

*CXCR4* plays an important role in controlling both adaptive and innate immune reactions. It controls leucocyte trafficking and movement between and among peripheral tissues, contributes to lymph node organization, and ultimately maintains the priming of T cells by influencing the development and stabilization of the immune system. *CXCR4*, in conjunction with the glycoprotein CD11b, facilitates neutrophil relocalization via lymphatic capillaries after bacterial infection [16].

*CXCR4* plays a significant role in a number of illnesses, such as cancer, autoimmune disorders, and immunodeficiency conditions. In fact, *CXCR4* expression serves as a prognostic indicator for a number of human malignancies, such as pancreatic, ovarian, and breast adenocarcinomas. Targeting *CXCR4* and its secondary location in tumor development is one of the major processes in the spread of cancer [17].

It is worth noting that understanding the possible involvement of host genetics in COVID-19 might pave the way for clinical studies and customized treatment, enabling us to contribute to a greater extent to the battle over the present coronavirus pandemic. The present study aimed to evaluate the association between the rs2228014 polymorphism in the *CXCR4* gene and the severity of COVID-19, which, to our knowledge, has not been previously reported. Moreover, studying the *CXCR4* receptor in COVID-19 patients could enhance our knowledge about the mechanisms of SARS-CoV-2 infection and the process of cytokine storm induced by the virus itself.

## Materials and methods

### Study design and participants

This cross-sectional study was conducted on 156 mild or moderate and 144 severe or critical adult male and female Egyptian patients admitted to Assiut University Quarantine Hospital from June 2022 to September 2022 during the emergence of Omicron variant of SARS-CoV-2. The diagnosis of COVID-19 depends on positive results of nasal or pharyngeal swabs obtained by reverse transcription polymerase chain reaction (RT‒PCR), in accordance with the WHO guidelines [18]. The included participants were divided into two groups according to their clinical manifestations: group I included individuals with mild or moderate symptoms, and group II included individuals with severe or critical symptoms.

### The inclusion criteria were as follows

All adult (aged > 18 years) Egyptian patients who were diagnosed with COVID-19 and had complete blood tests on the first day of admission were included in the study. The exclusion criteria were as follows: (1) non-Egyptian patients; (2) patients aged less than 18 years and those aged more than 85 years; (3) patients with incomplete medical records, missing clinical and hematology data and without available clinical outcome data; (4) patients who were referred from other centers after several days of hospitalization without an attached admission complete blood count (CBC) in the first center; and (5) patients with medical conditions of cancer, human immunodeficiency virus (HIV), obstructive lung disease, asthma or autoimmune diseases.

Fundamental demographic details such as age, sex, race, and associated conditions were gathered. The length of stay, the rate of admission to the intensive care unit (ICU), the length of ICU stay, the duration of mechanical ventilation, and the ultimate disposition were all documented. The researchers computed the median CRP concentration during the whole hospitalization stay for each patient within the first 7 days of hospitalization, as well as the peak and slope of the CRP change. Our main endpoint was predicted using these three CRP values. The primary outcome was all-cause in-hospital death. The secondary outcomes were hospital duration of stay, intensive care unit length of stay, and duration of mechanical ventilation.

The laboratory procedures performed on the patients included complete blood count (CBC), CRP, D-dimers, ferritin, and inflammatory marker assessments. Age, sex, cigarette smoking status, medical history, and prescription history were among the clinical and medical data that were extracted from the patients’ files. All patients were treated in accordance with the treatment protocol of the Egyptian Ministry of Health for COVID-19 [19].

### Study variables

Regarding the severity of COVID-19, mild or moderate cases included those with a large number of symptoms except dyspnea and with an oxygen saturation (SpO2) greater than 94%. Severe or critical cases were characterized by the incidence of one or more of the following: (a) an oxygenation index less than 300 mm Hg (arterial partial pressure of oxygen/inspired oxygen fraction, PaO2/FiO2), (b) a respiratory rate > 30 breaths/min, and (c) a pulse oximeter SpO2 < 93%.

Since pulmonary lesions cannot be ruled out by a routine chest X-ray, especially in patients with no symptoms and mild cases, all participants underwent chest CT scans.

*CXCR*4 rs2228014 selection: this Single nucleotide polymorphism (SNP) was selected following two criteria: (i) focused on SNPs that were associated with chest diseases, viral infection, and immunodeficiency to be related to COVID-19. *CXCR*4 rs2228014 is complicated in various illnesses like pulmonary disease, human immunodeficiency virus (HIV), human papillomairus (HPV), warts disease, pulmonary artery hypertension (PAH), and cancer and (ii) SNP was determined as it has been separately discussed no less than two times and the reported values for odds ratio were ≤ 0.9 for protective and ≥ 1.1 risk alleles. SNP was determined by literature search and a confirmed data base for SNP [20].

### Specimen collection

For real-time PCR analysis of the rs2228014 polymorphism in the *CXCR4* gene, two ml of venous blood was drawn from each patient through venipuncture and placed in a vacutainer EDTA tube. Blood samples were kept frozen at -20 °C until DNA was extracted.

### Genotyping analysis

#### DNA extraction

A genomic DNA purification kit supplied by Thermo Fisher Scientific was used for DNA isolation from the blood of COVID-19 patients according to the main protocol. Catalog number: K0512.

#### Genotyping of *CXCR4* rs2228014

The master mix used for DNA amplification was Genotyping TaqPath 1-Step Multiplex (Thermo Fisher; Cat. No. A28521). The amplification steps were as follows: enzyme activation for 10 min at 95 °C, followed by 35 cycles of denaturation for 15 s at 95 °C, annealing for 1 min at 60 °C, and elongation for 1 min at 72 °C. A TaqMan ready-made SNP assay was used (Thermo Fisher; Catalog number. 4,351,379) with a context sequence [VIC/FAM]:

GCCTCTGACTGTTGGTGGCGTGGAC[A/G]ATGGCCAGGTAGCGGTCCAGACTGA. The following PCR mixtures (20 µL) were used: Master Mix (5 µL), extracted DNA (3 µL), SNP assay (0.5 µL), and distilled water (11.5 µL). Real-time PCR equipment (Applied Biosystems 7500) was utilized.

### Statistical analysis

The data were processed and analyzed using the IBM SPSS software package version 20.0. (Armonk, NY: IBM Corp.) [21]. A goodness-of-fit (χ2) is used to estimate Hardy–Weinberg equilibrium (HWE). Qualitative data are presented as numbers and percentages. The Kolmogorov‒Smirnov test was used to verify the normality of the distribution. The quantitative data are presented as the range (minimum and maximum), mean, and standard deviation. The chi-square test was used to compare categorical variables between different groups. Student’s t test was used for normally distributed quantitative variables to compare two study groups. The Mann‒Whitney test was used for abnormally quantitative variables to compare two study groups.

Receiver operating characteristic (ROC) curve analysis was used to assess the predictive capacity of CRP for severity and to determine the optimal cutoff value, sensitivity, and specificity of CRP for all-cause disease severity. These results are reported as the area under the curve (AUC) and 95% confidence intervals (CIs). The odds ratios were calculated by comparing the occurrence of severe disease in two different groups of independent variables. For multivariable logistic regression, a 95% confidence interval for an adjusted odds ratio (AOR) was calculated, and variables with a p value ≤ 0.05 were considered to be significantly associated with disease outcome and severity.

## Results

The age of the patients ranged from 36 to 85 years, with a mean value of 64.31 ± 11.002 years. The study included 169 male patients (56.3%) and 131 female patients (43.7%).

### Demographic and clinical characteristics of COVID-19 patients

Table 1 shows the comparison according to demographic data and clinical findings of COVID-19 patients. The vaccination rates were significantly greater in the mild or moderate group than in the severe or critical group (*p* value 0.017). Hypertension, renal disease and diabetes mellitus (DM) were more prevalent in the severe or critical group than in the mild or moderate group (*p* values < 0.001, < 0.001, and 0.003, respectively).

**Table 1:** Comparison according to demographic characteristics and clinical findings of COVID-19 patients

	Mild or moderate (*n* = 156)		Severe or critical (*n* = 144)		OR (95%CI)	*p* value*
	No.	%	No.	%		
**Age (years)**						
≤ 65	82	52.6	62	43.1	1.47 (0.93–2.31)	&0.107
> 65	74	47.4	82	56.9		
Median (Range)	65.00 (36–82)		67.00 (37–85)		------	§ 0.634
**Sex**						
Male	91	58.3	78	54.2	1.19 (0.75–1.87)	&0.486
Female	65	41.7	66	45.8		
**Smoking**						
No	93	59.6	89	61.8	0.91 (0.57–1.45)	&0.724
Yes	63	40.4	55	38.2		
**Vaccination** **(AstraZeneca or** ***Pfizer*** **)**						
No	38	24.4	54	37.5	0.54 (0.33–0.88)	&0.017*
Yes	118	75.6	90	62.5		
**Comorbid diseases**						
None	69	44.2	22	15.3	0.23 (0.13–0.40)	&<0.001^*^
Hypertension	37	23.7	70	48.6	3.04 (1.86–4.98)	&<0.001^*^
Liver disease	11	7.1	6	4.2	0.57 (0.21–1.59)	&0.325
Renal disease	10	6.4	29	20.1	3.68 (1.72–7.87)	&<0.001^*^
DM	29	18.6	49	34.0	2.26 (1.33–3.84)	&0.003^*^
Thyroid disease	7	4.5	5	3.5	0.77 (0.24–2.47)	&0.772
Heart disease	8	5.1	5	3.5	0.67 (0.21–2.08)	&0.577

§Mann‒Whitney test. &Fisher’s exact test. *Significant. DM: diabetes mellitus. OR: odds ratio

CI: confidence interval. *p* values < 0.05 indicate statistical significance

### Comparison according to genetic (*CXCR4* rs2228014) findings

Table 2 shows that comparisons according to genetic (*CXCR4* rs2228014) findings of COVID-19 patients with mild or moderate and severe or critical symptoms revealed no significant differences between the two groups regarding allelic and genotypic frequencies. Patients with the GG genotype had a 1.86-fold greater risk of developing severe or critical COVID-19 than did those with the AA genotype. Patients with the G allele had a 2.11-fold greater risk of developing severe or critical COVID-19 than patients with the A allele.

**Table 2:** Genetic (CXCR4 rs2228014) findings of COVID-19 patients

CXCR4 rs2228014	Mild or moderate (*n* = 156)		Severe or critical (*n* = 144)		OR (95%CI)	*p* value
	No.	%	No.	%		
**Genotypes**						
GG	137	87.8	134	93.1	1.86(0.83–4.14)	&0.170
GA	16	10.3	10	6.9	0.65(0.29–1.49)	&0.412
AA	3	1.9	0	0	0.52(0.46–0.58)	&0.249
GA + AA	19	12.2	10	6.9	0.54 (0.24–1.20)	&0.170
P _HWE_	< 0.001		< 0.001			
**Alleles**						
G	290	92.9	278	96.5	2.11(0.98–4.53)	&0.068
A	22	7.1	10	3.5		

&Fisher’s exact test. HWE = Hardy-Weinberg equilibrium. OR: odds ratio. CI: confidence interval. *p* values < 0.05 indicate statistical significance

The frequencies of genotypes variants in both studied groups were statistically significant different than the expected according to HWE with *p* value < 0.001. This can be explained by different reasons; sample size, gene drift, selection criteria, different ethnic background of selected individuals or genetic flow [22].

### Patient demographic data, comorbidities, and symptoms according to *CXCR4* rs2228014 genotype

Table 3 shows a comparison between genotypes (*CXCR4* rs2228014) according to demographic data, comorbidities, and symptoms. In addition, there was a significant difference between the genotypes with regard to myalgia, where it was more prevalent in the combined GA + AA and AA genotypes than in the GG and GA, GG genotypes, respectively (*p* values of 0.018 and 0.019, respectively).

**Table 3:** Comparisons between genotypes (CXCR4 rs2228014) according to demographic data, comorbidities, and symptoms

	CXCR4 rs2228014							
	GG (***n = 271)***	GA + AA (***n = 29)***	OR (95%CI)	*p*value*	GG (***n = 271)***	GA (***n = 26)***	AA (***n = 3)***	*p*value*
	No. (%)	No. (%)			No. (%)	No. (%)	No. (%)	
**Age (years)**								
≤ 65	133(49.1)	11(37.9)	1.58 (0.72–3.46)	&0.329	133(49.1)	9(34.6)	2(66.7)	&0.300
> 65	138(50.9)	18(62.1)			138(50.9)	17(65.4)	1(33.3)	
Median (Range)	66(36–93)	70(47–82)	------	§ 0.107	66 (36–93)	70.5 (47–82)	62 (58–68)	§ 0.155
**Sex**								
Male	153(56.5)	16(55.2)	1.05 (0.49–2.28)	&1.000	153(56.5)	13(50.0)	3(100)	&0.253
Female	118(43.5)	13(44.8)			118(43.5)	13(50.0)	0(0)	
**Smoking**								
No	162(59.8)	20(69.0)	0.67 (0.29–1.52)	&0.425	162(59.3)	19(73.1)	1(33.3)	&0.258
Yes	109(40.2)	9(31.0)			109(40.2)	7(26.9)	2(66.7)	
**Vaccination** **(AstraZeneca or** ***Pfizer***								
No	80(29.5)	12(41.4)	0.59 (0.27–1.30)	&0.206	80(29.5)	10(38.5)	2(66.7)	&0.254
Yes	191(70.5)	17(58.6)			191(70.5)	16(61.5)	1(33.3)	
**Comorbid diseases**								
None	86(31.7)	5(17.2)	0.45 (0.17–1.21)	&0.137	86(31.7)	5(19.2)	0(0)	&0.215
Hypertension	96(35.4)	11(37.9)	1.11 (0.51–2.46)	&0.839	96(35.4)	10(38.5)	1(33.3)	&0.950
Liver disease	15(5.5)	2(6.9)	1.26 (0.27–5.83)	&0.674	15(5.5)	1(3.8)	1(33.3)	&0.107
Renal disease	35(12.9)	4(13.8)	1.08 (0.35–3.29)	&0.778	35(12.9)	4(15.4)	0(0)	&0.748
DM	69(25.5)	9(31.0)	1.32 (0.57–3.03)	&0.509	69(25.5)	7(26.9)	2(66.7)	&0.268
Thyroid disease	10(3.7)	2(6.9)	1.93 (0.40–9.28)	&0.326	10(3.7)	2(7.7)	0(0)	&0.572
Heart disease	11(4.1)	2(6.9)	1.75 (0.37–8.31)	&0.364	11(4.1)	2(7.7)	0(0)	&0.640
**Symptoms**								
Fever	67(24.7)	6(20.7)	0.79 (0.31–2.03)	&0.820	67(24.7)	6(23.1)	0(0)	&0.604
Sore throat	102(37.6)	8(27.6)	0.63 (0.27–1.48)	&0.318	102(37.6)	7(26.9)	1(33.3)	&0.552
Dry cough	145(53.5)	16(55.2)	1.07 (0.50–2.31)	&1.000	145(53.5)	15(57.7)	1(33.3)	&0.715
Headache	62(22.9)	7(24.1)	1.07 (0.44–2.63)	&0.820	62(22.9)	6(23.1)	1(33.3)	&0.912
Dyspnea	134(49.4)	10(34.5)	0.54 (0.24–1.20)	&0.170	134(49.4)	10(38.5)	0(0)	&0.139
Diarrhea	20(7.4)	1(3.4)	0.45 (0.06–3.47)	&0.705	20(7.4)	1(3.8)	0(0)	&0.711
Myalgia	105(38.7)	18(62.1)	2.59 (1.18–5.69)	&0.018*	105(38.7)	15(57.7)	3(100)	&0.019*
Fatigue	150(55.4)	15(51.7)	0.86 (0.40–1.86)	&0.845	150(55.4)	14(53.8)	1(33.3)	&0.742
Nausea	40(14.8)	4(13.8)	0.92 (0.31–2.80)	&1.000	40(14.8)	3(11.5)	1(33.3)	&0.594
Vomiting	15(5.5)	0(0)	0.90 (0.86–0.93)	&0.376	15(5.5)	0(0)	0(0)	&0.430
Anosmia	77(28.4)	8(27.6)	0.96 (0.41–2.26)	&1.000	77(28.4)	7(26.9)	1(33.3)	&0.969
Gustatory dysfunction	62(22.9)	7(24.1)	1.07 (0.44–2.63)	&0.820	62(22.9)	6(23.1)	1(33.3)	&0.912
Dysarthria	9(3.3)	0(0)	0.90 (0.87–0.94)	&1.000	9(3.3)	0(0)	0(0)	&0.609
**Clinical course**								
Mild or moderate illness	137(50.6)	19(65.5)	0.54 (0.24–1.20)	&0.170	137(50.6)	16(61.5)	3(100)	&0.139
Severe or critical illness	134(49.4)	10(34.5)			134(49.4)	10(38.5)	0(0)	

§Mann‒Whitney test. &Fisher’s exact test. *Significant. p values < 0.05 indicated statistical significance. *Significant. OR: odds ratio. CI: confidence interval. DM: diabetes mellitus

### Laboratory evaluation and CT findings according to *CXCR4* rs2228014 genotype

Table 4 shows a comparison between genotypes according to laboratory evaluation and CT findings. There was a significant difference between genotypes (GG vs. GA + AA) with regard to the median lymphocyte count, where the median lymphocyte count was significantly lower in the combined GA + AA genotype group than in the GG genotype group (*p* value 0.031). The leucocyte count was significantly lower in the combined GA + AA genotype and the AA genotype than in the GG and the GA and GG genotypes (p values of 0.007 and 0.004, respectively).

**Table 4:** Comparisons between genotypes (CXCR4 rs2228014) according to laboratory evaluation and CT findings (all units are in accordance with the international standard units and reference ranges) [46]

	CXCR4 rs2228014							
	GG (***n = 271)***	GA + AA (***n = 29)***	OR (95%CI)	*p*value*	GG (***n = 271)***	GA (***n = 26)***	AA (***n = 3)***	*p*value*
	No. (%)	No. (%)			No. (%)	No. (%)	No. (%)	
**Laboratory evaluation**								
**lymphocyte count** (×10^9^/L) (1.0–3.0)								
< 1.0	72(26.6)	12(41.4)	-----	#0.154	72(26.6)	10(38.5)	2(66.7)	#0.308
1.0–4.0	169(62.4)	16(55.2)			169(62.4)	15(57.7)	1(33.3)	
> 4.0	30(11.1)	1(3.4)			30(11.1)	1(3.8)	0(0)	
Median (Range)	1.50 (0.4–5.8)	1.20 (0.4–5.8)	------	§ 0.031*	1.50 (0.4–5.8)	1.20 (0.5-5.0)	0.70 (0.6–1.6)	§ 0.082
**Leucocyte (×10** ^**9**^ **/L)** **(**4.0–11.0)								
< 4.0	54(19.9)	7(24.1)	-----	#0.007*	54(19.9)	6(23.1)	1(33.3)	#0.040*
4.0–10.0	122(45.0)	20(69.0)			122(45.0)	18(69.2)	2(66.7)	
> 10.0	95(35.1)	2(6.9)			95(35.1)	2(7.7)	0(0)	
Median (Range)	8.10 (1.8–16.2)	6.50 (2.0-12.8)	------	^0.010*	8.10 (1.8–16.2)	6.70 (2.0-12.8)	5.70 (2.0-6.5)	^0.026*
**Platelets**(×10^3^/L) **(150–450)**								
≤ 100	40(14.8)	4(13.8)	1.08 (0.36–3.28)	&1.000	40(14.8)	4(15.4)	0(0)	&0.768
> 100	231(85.2)	25(86.2)			231(85.2)	22(84.6)	3(100)	
Median (Range)	245.0 (72–420)	240.5 (71–402)	------	§ 0.555	245.00 (72–420)	237.00 (71–402)	240.00 (236–247)	§ 0.840
**CRP level (mg/L)** **(less than 6.0)**								
≤ 5	46(17.0)	4(13.8)	1.28 (0.42–3.85)	&0.798	46(17.0)	4(15.4)	0(0)	&0.723
> 5	225(83.0)	25(86.2)			225(83.0)	22(84.6)	3(100)	
Median (Range)	48 (4-768)	48 (4-384)	------	§ 0.437	48.00 (4-768)	48.00 (4-384)	48.00 (12–48)	§ 0.663
**Ferritin (µg/L)** **(150–300)**								
≤ 300	90(33.2)	10(34.5)	0.95 (0.42–2.12)	&1.000	90(33.2)	10(38.5)	0(0)	&0.405
> 300	181(66.8)	19(65.5)			181(66.8)	16(61.5)	3(100)	
Median (Range)	396.0 (10-2500)	533.0 (107–1370)	------	§ 0.996	396.00 (10-2500)	550.00 (107–1370)	352.00 (344–1100)	§ 0.929
D-dimers **levels (µg/L)** **(< 0.5)**								
< 0.5	107(39.5)	13(44.8)	------	#0.785	107(39.5)	12(46.2)	1(33.3)	#0.619
0.5-1.0	83(30.6)	9(31.0)			83(30.6)	7(26.9)	2(66.7)	
> 1.0	81(29.9)	7(24.1)			81(29.9)	7(26.9)	0(0)	
Median (Range)	0.70 (0.1–3.3)	0.60 (0.1–2.4)	------	§ 0.370	0.70 (0.1–3.3)	0.65 (0.1–2.4)	0.60 (0.2–0.7)	§ 0.625
**Imaging**								
**CT findings**								
Normal	26(9.6)	1(3.4)	0.34 (0.04–2.58)	&0.492	26(9.6)	1(3.8)	0(0)	&0.533
Bilateral GGO	181(66.8)	24(82.8)	2.39 (0.88–6.46)	&0.094	181(66.8)	22(84.6)	2(66.7)	&0.175
Pneumonic consolidation	99(36.5)	8(27.6)	0.66 (0.28–1.55)	&0.417	99(36.5)	6()23.1	2(66.7)	&0.208

^Independent t test. #Chi square test. §Mann‒Whitney test. &Fisher’s exact test. *p* values < 0.05 indicated statistical significance. *Significant. OR: odds ratio. CI: confidence interval. CRP: C-reactive protein, CT: computed tomography, GGO: ground-glass opacity, L: liter, mL: millileter, mg/L: milligram/Liter, µg/L: microgram/Liter

### Treatment and outcomes according to *CXCR4* rs2228014 genotype

As shown in Table 5, there was a significant difference between the genotypes (GG vs. GA vs. AA) regarding antibiotic treatment, where antibiotic use was significantly lower in the AA genotype than in the GA and GG genotypes (*p* value < 0.001). There were no significant differences regarding other factors (*p* value > 0.05).

**Table 5:** Comparison between genotypes (CXCR4 rs2228014) according to treatment and outcomes

	CXCR4 rs2228014							
	GG (***n = 271)***	GA + AA (***n = 29)***	OR (95%CI)	*p*value*	GG (***n = 271)***	GA (***n = 26)***	AA (***n = 3)***	*p*value*
	No. (%)	No. (%)			No. (%)	No. (%)	No. (%)	
Antibiotic	261(96.3)	27(93.1)	0.52 (0.11–2.48)	&0.326	261(96.3)	26(100)	1(33.3)	&<0.001*
Antifungal	16(5.9)	2(6.9)	1.18 (0.26–5.41)	&0.689	16(5.9)	1(3.8)	1(33.3)	&0.123
Antiviral	207(76.4)	25(86.2)	1.93 (0.65–5.76)	&0.349	207(76.4)	22(84.6)	3(100)	&0.406
Glucocorticoids	156(57.6)	20(69.0)	1.64 (0.72–3.73)	&0.321	156(57.6)	18(69.2)	2(66.7)	&0.494
Clexane	84(31.0)	8(27.6)	0.85 (0.36–1.99)	&0.833	84(31.0)	7(26.9)	1(33.3)	&0.907
**Oxygen therapy**								
None	68(25.1)	5(17.2)	0.62 (0.23–1.69)	&0.495	68(25.1)	5(19.2)	0(0)	&0.492
Nasal cannula	71(26.2)	7(24.1)	0.90 (0.37–2.19)	&1.000	71(26.2)	7(26.9)	0(0)	&0.585
Mask oxygen	136(50.2)	14(48.3)	0.93 (0.43–1.99)	&1.000	136(50.2)	12(46.2)	2(66.7)	&0.782
Invasive mechanical ventilation	59(21.8)	5(17.2)	0.75 (0.27–2.05)	&0.811	59(21.8)	5(19.2)	0(0)	&0.633
**Outcomes**								
Home management	69(25.5)	9(31.0)	1.31 (0.57–3.03)	&0.509	69(25.5)	7(26.9)	2(66.7)	&0.268
Hospitalization without ICU	68(25.1)	10(34.5)	1.57 (0.70–3.54)	&0.272	68(25.1)	9(34.6)	1(33.3)	&0.548
ICU	134(49.4)	10(34.5)	0.54 (0.24–1.20)	&0.170	134(49.4)	10(38.5)	0(0)	&0.139
Death	36(13.3)	3(10.3)	0.75 (0.22–2.62)	&1.000	36(13.3)	3()11.5	0(0)	&0.772
**Duration of ICU stay (days)**								
Median(Range)	7.0(1–13)	7.50 (1–13)	------	§ 0.883	7.00 (1–13)	7.50 (1–13)	----	§ 0.883
**Duration of in-hospital stay (days)**								
Median(Range)	7.0(1–15)	8.50 (4–15)	------	§ 0.166	7.00 (1–15)	8.00 (4–15)	5.00 (5–15)	§ 0.093
**Duration of recovery (days)**								
Median (Range)	15(2–48)	14.50 (2–48)	------	§ 0.501	15.00 (2–48)	14.00 (2–46)	35.00 (13–48)	§ 0.332

§Mann‒Whitney test. &Fisher’s exact test. *Significant. *p* values < 0.05 indicated statistical significance. *Significant. OR: odds ratio. CI: confidence interval. ICU: intensive care unit

### ROC curve, cutoff value for the sensitivity and specificity of the CRP level according to clinical stage

**Fig. 1:**
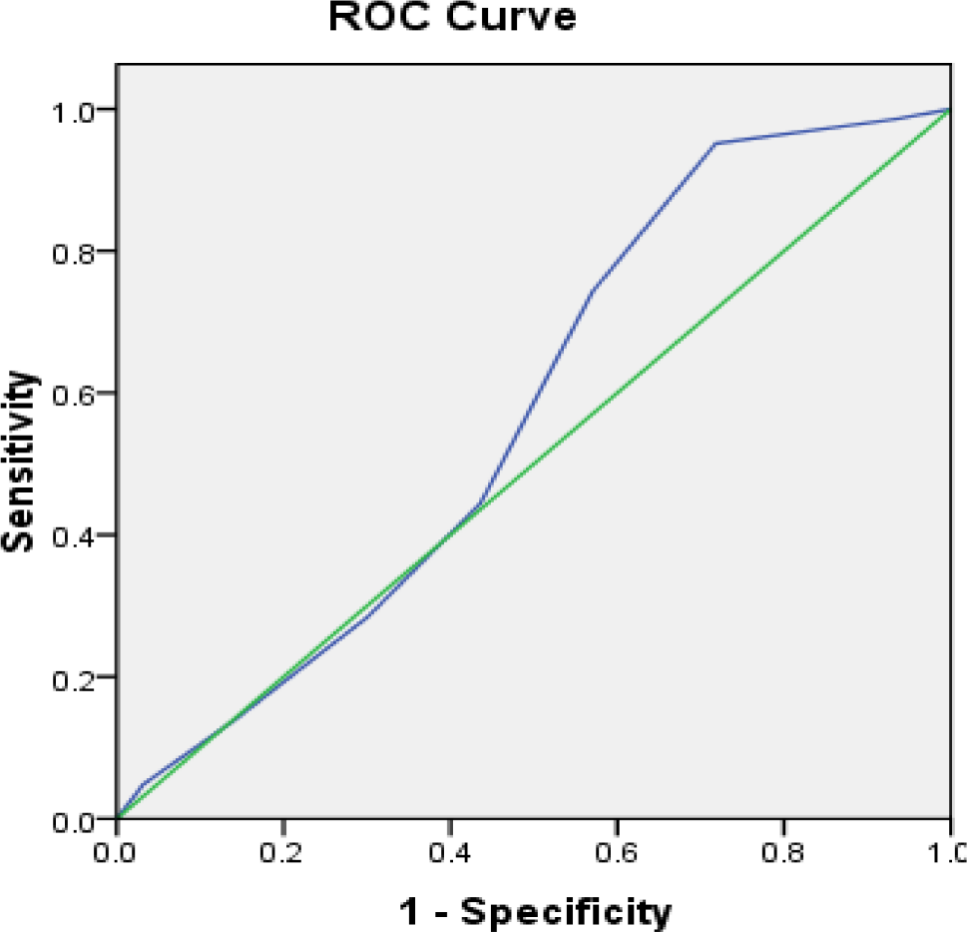
ROC curve of CRP levels according to the clinical stage of COVID-19

The ROC curve analysis showed CRP was a poor classifier of COVID-19 clinical stage; mild/moderate vs. severe/critical (AUC = 0.574, p value = 0.028 & 95% CI = 0.508–0.639). It clarified that the cut value of 30 mg/L had a sensitivity = 74.3% & Specificity = 42.9% (Fig. 1).

## Discussion

COVID-19 has an incubation period of 3 to 7 days globally, with approximately 80% of infections being moderate or asymptomatic, 15% being severe and requiring oxygen, and 5% being critical infections that require breathing. Numerous studies with various designs have attempted to clarify the relationship between certain host genetic variations and the severity of clinical illness or susceptibility to SARS-CoV-2 infection [23, 24].

The present study is a cross-sectional assessment of the genetic regarding the clinical characteristics of 156 mild or moderate and 144 severe or critical COVID-19 patients at Assiut University Quarantine Hospital during the period from June to September 2022. No significant differences between severity groups were found in the allelic or genotypic frequencies of *CXCR4* rs2228014. However, the exclusive presence of the AA genotype in mild or moderate cases suggests its potential protective role. Additionally, significant differences in myalgia presentation, leukocyte counts and antibiotic use, were observed among different genotypes.

There were no significant differences in sex, age, or smoking status between the mild or moderate group and the severe or critical group. A significant difference between the two groups was found regarding vaccination, which was more frequent in the mild or moderate group than in the severe or critical group. COVID-19 vaccination has effectively lowered the incidence of infections, severity, hospitalization, and death [25]. In agreement with the present results, a study by Tenforde et al., 2021 which was conducted on 4513 adult hospitalized COVID-19 patients, concluded that vaccination could decrease disease severity [26].

Despite the wide range of inconsistent findings from earlier studies, the present study independently evaluated comorbid diseases such as diabetes mellitus, hypertension, renal disease, heart disease, and thyroid disease among mild or moderate and severe or critical groups to better understand how these comorbid diseases could influence COVID-19 outcomes. There was a significant correlation between mild or moderate and severe or critical groups regarding hypertension, renal disease, and diabetes mellitus, where these comorbid diseases were more frequent in severe or critical cases than in mild or moderate cases. An observational study conducted by Taoufiq et al., 2021 on 567 COVID-19 patients indicated that severe or critical patients had more comorbid diseases, including diabetes and hypertension, than mild or moderate patients did [27].

Both type 1 and type 2 diabetes are characterized by chronic and sustained immunological dysregulation. There are significant alterations in phagocytic activity and cytokine production in both types and increased levels of proinflammatory mediators [28]. Diabetic patients may have higher ACE2 expression in their lungs, which may indicate a greater propensity for SARS-CoV-2 binding and subsequent replication. This might then trigger more ACE2 upregulation and pulmonary edema [29].

Hypertension may worsen COVID-19 by facilitating viral contact with host cells. Individuals with hypertension have weakened initial immunity against SARS-CoV-2 infection due to an increased innate immune response and persistent inflammation [30].

In addition, the frequencies of the AA genotype and the A allele of *CXCR4* rs2228014 were not significantly different between mild or moderate patients and severe or critical patients. However, the presence of the AA genotype in the mild or moderate group and its absence in the severe or critical group and the overexpression of the A allele in the mild or moderate group compared with the severe or critical group may indicate the protective effect of rs2228014 against disease severity. According to this theory’s evidence, the A allele may decrease *CXCR4* transcription in comparison to the G allele, which would reduce the effectiveness of the SARS-CoV-2 pathway by impeding the ability of SARS-CoV-2 to bind to the *CXCR4* receptor.

The G allele was the predominant in both our studied groups with low frequency of A allele this may explain that A allele cannot reach to a statistical significance for the risk of severity of COVID-19. This was compatible with the results of 1000 Genomes_30x for the frequency of rs2228014, as the genome results clarified that G allele represented the highest volume in all populations with a frequency of 0.9393, 0.9866, 0.9542, 0.9193, 0.8444 & 0.971 in the study wide group, subgroups of African, Europe, South Asian, and American respectively [31].

In accordance with the findings of the present study, a Turkish study by Dalan et al., 2020 involving 61 dementia patients aimed at evaluating the role of *CXCR4* rs2228014 in dementia susceptibility revealed that the *CXCR4* T allele may be associated with a decreased risk of dementia [32]. In contrast to the present study results, Okuyama et al., 2022 reported that *CXCR4* rs2228014 is a risk factor for HPV infection, where the presence of this SNP increases vulnerability to HPV infection [33].

A study by Matsusaka et al., 2015 conducted to evaluate the effect of *CXCR4* variants on patients with metastatic colorectal cancer revealed that patients with rs2228014 AA have a significantly shorter progression-free survival (PFS) than patients with the GG genotype (10.5 vs. 9.6 months, HR (95% CI) 1.40 (1.02–1.93), *p* = 0.035) [34].

The comparison between genotypes (*CXCR4* rs2228014), according to demographic data, comorbidities, and symptoms, revealed a significant difference between the genotypes with regard to myalgia, where it was more prevalent in the combined GA + AA and AA genotypes than in the GG and the GA and GG genotypes.

The comparison between genotypes according to laboratory evaluation and CT findings revealed a significant difference between genotypes (GG vs. GA + AA) with regard to the median lymphocyte count, where the median lymphocyte count was significantly lower in the combined GA + AA genotype group than in the GG genotype group. The leucocyte count was significantly lower in the combined GA + AA genotype and the AA genotype than in the GG and the GA and GG genotypes, respectively.

The present data revealed a significant difference between genotypes (GG vs. GA vs. AA) with regard to antibiotic treatment, where antibiotic resistance was significantly lower in the AA genotype than in the GA and GG genotypes.

*CXCR4* plays a central role in virus elimination through the activation of MAPK/ERK (mitogen-activated protein kinase/extracellular signal-regulated kinase signaling pathway) and PI3K/Akt (lipid kinase phosphoinositide-3-kinase signaling pathway), which leads to neutrophil activation and virus elimination [35].

The expression of *CXCR4* is upregulated in severely hypoxemic COVID-19 patients. During hypoxia, hypoxia-inducible factor 1-alpha (HIF1α) is upregulated, which in turn leads to the upregulation of immune-related genes such as the genes encoding the chemokine receptors *CXCR2*, *CXCR4*, and *CXCR1*. *CXCR4* has proinflammatory properties. It is thought that the expression of *CXCR4*, which is prevalent in COVID-19 patients, may serve as a prognostic indicator for ARDS and lung damage [36].

*CXCR4* inhibition has been proposed to have beneficial effects on both the prevention and management of acute respiratory distress syndrome and related cytokine storms, pulmonary fibrosis, and imbalanced angiogenesis in SARS-CoV-2-infected lungs. Balixafortide (a powerful, effective antagonist of CXCR4) showed a dose-dependent cell protective effect in a SARS-CoV-2-induced cytopathic effect test (CPE) in vitro. Balixafortide therapy resulted in considerably fewer infectious SARS-CoV-2 particles on day 4 after infection, as well as a decreased overall viral load on day 10 [37].

The present study revealed that the CRP was significantly greater in the severe or critical group than in the mild or moderate group. These data agree with those of Muruh et al., 2021 who reported a significant increase in CRP in severe COVID-19 patients [38]. These data are in agreement with those of Rokni et al., 2022, who reported a statistically significant difference in CRP between patients with non-severe and severe disease [39].

The present results showed that the severity of patients can be predicted at a cutoff value of 30, with a sensitivity of 74.3% and a specificity of 42.9%. A correlation between CRP and disease severity was shown by Wang et al., 2020 using logistic regression analysis revealed that CRP was substantially linked with worsening of non-severe to severe COVID-19, with an area under the curve of 0.844 (95% confidence range, 0.761–0.926) and an appropriate threshold value of 26.9 mg/l [40].

CRP is an acute-phase protein that acts as an early indicator of infection or inflammation. In general, it is substantially greater in bacterial illnesses. CRP levels, during infectious or inflammatory disease states, can activate the immune system’s classical complement cascade and influence the activity of phagocytic cells, suggesting that CRP functions in the opsonization of infectious pathogens and dead or dying cells. The exact effect of CRP on COVID-19 is unknown, although it has been reported that its level can be utilized for the early detection of pneumonia and the assessment of severe lung infectious disorders [41].

The overproduction of inflammatory cytokines in patients with severe COVID-19 may be responsible for the high levels of CRP. In individuals with non-severe COVID-19, a high CRP level may be a useful early marker for predicting the probability of disease development and can aid healthcare professionals in early patient identification and treatment [42].

Cytokines play important roles in COVID-19 pathogenesis. However, little is known about the reason for and relevance of genetic differences related to immune system responses, often known as “immunogenetic profiling” [43]. The present findings could offer fresh knowledge of the many aspects influencing illness severity and the processes of cytokine storm syndrome that might have an impact on COVID-19 results and therapeutic approaches. In this regard, AMD3100, a *CXCR4* antagonist, can block HIV entry into cells once the virus binds to the cell surface [44]. It is used to downregulate the expression of *CXCR4*, which leads to the inhibition of the uncontrolled production of a variety of key inflammatory cytokines by monocytes that play a central role in the cytokine storm prompted by SARS-CoV-2 in patients with severe COVID-19 [45].

Based on the previously mentioned data, patients’ admission circumstances, such as vaccination status, comorbidities, and other abnormal indicators may indicate disease severity. These variables require additional exploration and should be taken into account for risk categorization. It has been discovered that COVID-19 has advanced quickly in several severely sick individuals. Close monitoring and quick treatment can therefore be crucial for patients at high risk and may assist in improving the results.

### Limitations

Performing this investigation faced some challenges. First, there were fewer patients for whom it was challenging to collect samples from severe instances, individuals with no symptoms, or patients who had recovered. Second, certain patients who had negative smear results or certain comorbidities that were not appropriate for the research were excluded from the analysis.

### Recommendations

The present study recommends the following: Investigating other SNPs in the *CXCR*4 gene or other related genes. Increasing the number of populations included in research might help reveal more about the interactions between genetic variations and various ethnic origins. More research on the underlying mechanisms via which mutations in the *CXCR*4 gene affect the severity of COVID-19. Increasing the sample size and diversifying the participant demographics may enhance the results’ generalizability. Examining the genetic variations in gene expression levels that might shed light on the ways in which COVID-19 severity is influenced by *CXCR*4 gene polymorphisms. The addition of genetic risk assessment to COVID-19 management protocols has the potential to improve treatment results and patient outcomes. Finding specific therapeutic targets that are impacted by *CXCR*4 gene variants may help create novel treatments for patient populations at high risk.

## Conclusion

The present study underscores the importance of genetic factors in determining COVID-19 outcomes and highlights the need for targeted approaches in the management of high-risk patients. Moreover, the AA genotype and A allele of *CXCR4* rs2228014 may confer protection against severe COVID-19. In addition, unvaccinated patients, patients with certain comorbidities, such as hypertension, renal disease, and diabetes mellitus, had an increased risk of having severe or critical disease rather than mild or moderate disease. These findings suggest potential targets for therapeutic intervention or risk stratification in COVID-19 management.

## Electronic supplementary material

Below is the link to the electronic supplementary material.

Supplementary Material 1

## Data Availability

The datasets generated and/or analyzed during the current study are available at the biosample depository, SubmissionID: SUB13604667 https://ncbi.nlm.nih.gov/subs/biosample/SUB13604667.

## Abbreviations

C: Degree Celsius
ACE 2: Angiotensin-converting enzyme II
ARDS: Acute respiratory distress syndrome
BCG: Bacille Calmette-Guérin
COPD: Chronic obstructive pulmonary disease
COVID-19: Coronavirus disease 2019
CT: Computed tomography
ICU: Intensive care unit
IFN-γ: Interferon-gamma
PCR: L: liter
mL: Millileter
mg/L: Milligram/Liter
µg/L: Microgram/Liter
RT‒PCR: Reverse transcription polymerase chain reaction
SARS-CoV-2: Severe acute respiratory syndrome coronavirus 2
SpO2: Tumor necrosis factor-alpha
TMPRSS2: Transmembrane protease serine 2
TNF-α: Oxygen saturation
WHO: World Health Organization
