# Supplementary material for: Potential protective association of the AA genotype and a allele of CXCR4 rs2228014 polymorphism with COVID-19 severity in adult egyptians
Source: BMC Infect Dis. 2024 Oct 15;24:1158. doi: 10.1186/s12879-024-09602-8 (PMC11479566; doi:10.1186/s12879-024-09602-8)
Supplement: Supplementary file 1 — Supplementary Material 1 [file 12879_2024_9602_MOESM1_ESM.docx]

**Supplementary data**

**Multivariate logistic regression of genotypes for clinical COVID19 Stage**

|  | | **P value** | **OR** | **95% C.I.** | |
| --- | --- | --- | --- | --- | --- |
|  |  |  |  | **Lower** | **Upper** |
| **Sever/critical COVID19** | **Genotyping of CXCR4**  GG/GA + AA | 0.206 | 0.588 | 0.258 | 1.340 |

**Multivariate logistic regression of genotypes for ICU admission**

|  | | **P value** | **OR** | **95% C.I.** | |
| --- | --- | --- | --- | --- | --- |
|  |  |  |  | **Lower** | **Upper** |
| **ICU admission** | **Genotyping of CXCR4**  GG/GA + AA | 0.206 | 0.588 | 0.258 | 1.340 |

**Multivariate logistic regression of genotypes for Death**

|  | | **P value** | **OR** | **95% C.I.** | |
| --- | --- | --- | --- | --- | --- |
|  |  |  |  | **Lower** | **Upper** |
| **Death** | **Genotyping of CXCR4**  GG/GA + AA | 0.764 | 0.825 | 0.235 | 2.898 |

**Multivariate logistic regression of genotypes for mechanical ventilation**

|  | | **P value** | **OR** | **95% C.I.** | |
| --- | --- | --- | --- | --- | --- |
|  |  |  |  | **Lower** | **Upper** |
| **Mechanical ventilation** | **Genotyping of CXCR4**  GG/GA + AA | 0.213 | 2.337 | 0.615 | 8.887 |
